# Prostate epithelial genes define therapy-relevant prostate cancer molecular subtype

**DOI:** 10.1038/s41391-021-00364-x

**Published:** 2021-04-26

**Authors:** Hyunho Han, Hyung Ho Lee, Kwibok Choi, Young Jun Moon, Ji Eun Heo, Won Sik Ham, Won Sik Jang, Koon Ho Rha, Nam Hoon Cho, Filippo G. Giancotti, Young-Deuk Choi

**Affiliations:** 1 Department of Urology, Urological Science Institute, Yonsei University College of Medicine, Seoul, Republic of Korea; 2 Center for Prostate Cancer, National Cancer Center, Goyang, Republic of Korea; 3 Department of Pathology, Yonsei University College of Medicine, Seoul, Republic of Korea; 4 Department of Genitourinary Oncology, The University of Texas MD Anderson Cancer Center, Houston, TX USA; 5 Department of Cancer Biology, The University of Texas MD Anderson Cancer Center, Houston, TX USA

**Keywords:** Cancer genetics, Prostate cancer

## Abstract

**Background and objectives:**

Transcriptomic landscape of prostate cancer (PCa) shows multidimensional variability, potentially arising from the cell-of-origin, reflected in serum markers, and most importantly related to drug sensitivities. For example, Aggressive Variant Prostate Cancer (AVPC) presents low PSA per tumor burden, and characterized by de novo resistance to androgen receptor signaling inhibitors (ARIs). Understanding PCa transcriptomic complexity can provide biological insight and therapeutic guidance. However, unsupervised clustering analysis is hindered by potential confounding factors such as stromal contamination and stress-related material degradation.

**Materials and methods:**

To focus on prostate epithelial cell-relevant heterogeneity, we defined 1,629 genes expressed by prostate epithelial cells by analyzing publicly available bulk and single- cell RNA sequencing data. Consensus clustering and CIBERSORT deconvolution were used for class discovery and proportion estimate analysis. The Cancer Genome Atlas Prostate Adenocarcinoma dataset served as a training set. The resulting clusters were analyzed in association with clinical, pathologic, and genomic characteristics and impact on survival. Serum markers PSA and PAP was analyzed to predict response to docetaxel chemotherapy in metastatic setting.

**Results:**

We identified two luminal subtypes and two aggressive variant subtypes of PCa: luminal A (Adipogenic/AR-active/PSA-high) (30.0%); luminal S (Secretory/PAP-high) (26.0%); AVPC-I (Immune-infiltrative) (14.7%), AVPC-M (Myc-active) (4.2%), and mixed (25.0%). AVPC-I and AVPC-M subtypes predicted to be resistant to ARI and have low PSA per tumor burden. Luminal A and AVPC-M predicted to be resistant to docetaxel and have high PSA/PAP Ratio. Metastatic PCa patients with high PSA/PAP ratio (>20) had significantly shorter progression-free survival than those with low ratio (≤20) following docetaxel chemotherapy.

**Conclusion:**

We propose four prostate adenocarcinoma subtypes with distinct transcriptomic, genomic, and pathologic characteristics. PSA/PAP ratio in advanced cancer may aid in determining which patients would benefit from maximized androgen receptor inhibition or early use of antimicrotubule agents.

## Introduction

Previous attempts to subtype PCa by transcriptomic variability, including ETS transcription-factor– based classifications and luminal/basal lineage models [1–3], was not able to provide additional clinical information beyond known risk factors [4]. Currently, therapeutic options for advanced PCa include AR signaling inhibitors (ARIs - abiraterone, enzalutamide, apalutamide), antimicrotubule agents (docetaxel, cabazitaxel), and immune therapy (sipuleucel-T). However, increasing evidences suggest intrinsically AR-independent tumors exist, characterized by neuroendocrine or small cell histology and mutations of multiple tumor suppressors *PTEN*, *TP53* or *RB1* [5–7]. PCa of intrinsic resistance to docetaxel has been reported [8], too. Therefore, an ideal PCa classification system should be able to determine for which tumors ARI, docetaxel, immunotherapy or other newly developing therapies can be offered.

PCa is characterized by multifocality or intratumoral heterogeneity [9, 10]; in addition, stromal contents (fibroblasts, endothelial cells, immune cells) can add further diversity. Therefore, it is likely that a tumor may be composed of more than two molecular subtypes that differ in the tumor cell, as well as tumor-microenvironment gene expression [11–13]. Whole-transcriptome analysis of tumor tissue is susceptible to those potential confounding factors when attempting to identify subtypes based on the tumor cell intrinsic heterogeneity.

For normal prostate tissue, single-cell analysis precisely defined epithelial-expressed genes and confirmed the existence of luminal, basal, or bipotential progenitor populations with specific anatomical locations and potential relevance to cancer characteristics such as AR independence [14–16]. We hypothesized that the PCa transcriptome can be interpreted based on their cell-of-origin of gene expression, especially considering therapeutic relevance. Using the single- cell RNA-seq data and an established deconvolution analysis tool, we developed a single-sample subtype classifier with proportion estimate (PE) for a given prostate tumor RNA-seq data. We report four transcriptomic subtypes with different predicted sensitivities to antimicrotubule agents and ARIs, and utility of serum biomarkers PSA and prostate-specific acid phosphatase (PAP) combination to select patients who will most likely benefit from each class of drugs.

## Materials and methods

### Prostate epithelial-expressed gene identification from single-cell and bulk RNA-seq data

We used *single-cell RNA-seq data* from Henry et al. [14] that used three human prostate specimens. Mapped read count data were downloaded from GEO (GSE117403) and aggregated using 10X Genomics Cell Ranger aggregate function. We followed the analysis pipeline of Henry et al and replicated differentially expressed gene (DEG) lists for luminal, basal, club-like, hillock-like, and neuroendocrine prostate epithelial cells. We chose overexpressed genes by those five epithelial cell populations in comparison to the rest epithelial and non-epithelial cells (Supplementary Table S1A).

We also analyzed the *bulk RNA-seq data* of corresponding fluorescence-activated cell sorting- isolated human prostate cell types from the same study [14]. We downloaded the fragments per kilobase million (FPKM)-value matrix of basal, luminal, and other epithelia, and fibromuscular stroma from GEO (GSE117271), selecting genes overexpressed by more than 5-fold by all epithelia, or by an epithelial subpopulation vs. the remainder of the epithelia (Supplementary Table S1B). We merged the DEG lists from single-cell and bulk datasets, and kept genes that mapped to an Entrez gene ID (Supplementary Table S1C).

### Consensus clustering of TCGA-PRAD (The Cancer Genome Atlas Prostate Adenocarcinoma) RNA-seq data

We downloaded annotated TCGA-PRAD gene expression, clinical, and genomic data from the UCSC Xena browser. RNA-seq by expectation maximization (RSEM) data containing mRNA expression levels of 550 samples were uploaded to the GenePattern Public server (cloud.genepattern.org). We used Consensus Clustering Module version 7.2 with parameters set at: Kmax = 15; resampling iterations = 20; clustering algorithm = self-organizing map; cluster by = columns; distance measure = Euclidean; resample = subsample with a proportion of 0.80; merge type = average; descent iterations = 2000; normalize type = row-wise; normalization iterations = 0. We used pre-calculated DNA purity (ABSOLUTE, CLONET) and RNA purity (ISOpure, DeMix purity) scores, AR activity score, AR mRNA and protein expression and survival data from the TCGA- PRAD dataset at the cBioPortal.

### RNA-seq data deconvolution and single-sample PE

We used CIBERSORT, a digital cytometry tool for deconvolution of heterogeneous tissues based on bulk mRNA-seq data [17]. RNA-seq read-normalized gene expression values (RSEM, RPKM, and FPKM for TCGA, CPC-GENE and DKFZ, and SU2C-PCF datasets, respectively) with Entrez gene ID and HUGO gene-symbol annotations were loaded as a “mixture” file. The gene signature was defined by the average gene expression values of prostate epithelial-expressed genes in the TCGA-PRAD dataset clusters predetermined by Consensus Clustering.

### In silico docetaxel and paclitaxel sensitivity test

To predict docetaxel sensitivity in PCa patients, we used a previously published gene expression signature associated with breast tumor response to docetaxel therapy, evaluated by the degree of reduction in tumor size [18]. Genes overexpressed (*n* = 13) and underexpressed (*n* = 43) in docetaxel responders were used as gene sets to run single-sample gene-set enrichment analysis (ssGSEA) loaded as a module in the GenePattern platform. The docetaxel responder score was calculated by subtracting the ssGSEA score of underexpressed genes from that of overexpressed genes. To predict paclitaxel sensitivity, we searched the cancer therapeutics response portal (CTRP v2, http://portal.broadinstitute.org/ctrp.v2.1) and selected genes whose expression correlated positively (Pearson *r* > 0.3) with cancer cell lines relative sensitivity to paclitaxel (1- area under curve value). All score values underwent z-score normalization.

### Docetaxel response analysis in the Yonsei University Health System (YUHS) database

The study design was approved by Severance Hospital Institutional Review Board (IRB #4-2020- 0812). The YUHS Big-Data team identified case of PCa patients who had serum PSA and PAP test results in a single sample to calculate the PSA and PAP ratio (PPR). The team also identified cases of metastatic castration-resistant prostate cancer (mCRPC) patients who (1) underwent at least three consecutive cycles of docetaxel-predisone chemotherapy, (2) had abdominopelvic CT and whole-body bone scan imaging before, during, and after chemotherapy to assess radiographic response, and (3) had serum PSA and PAP test results acquired within 30 days ahead of initial chemotherapy start date. Docetaxel response was measured using RECIST 1.1 criteria.

For serum PAP measurements, two assays were used: Prostatic Acid Phosphatase ran in IMMULITE 2000 (Siemens Genesis, result provided in ng/mL) and ACP Reagent (Roche) ran in TBA C8000 (Toshiba, result provided in U/L). We performed simple linear regression in samples of both data available (*n* = 975) and interpolated missing values from the equation (*n* = 78) (Supplementary Fig. S4f).

### Statistics and reproducibility

Statistical analyses were performed with GraphPad Prism version 8.4.3 (GraphPad, San Diego, CA, USA). *P* values were estimated using log-rank (Mantel–Cox) test for survival curve comparison, unless indicated otherwise. For analysis of correlation between drug-sensitivity scores and subtype PEs, Spearman *r* values and two-tailed *P* value were reported. For multiple comparisons, ANOVA and Kruskal–Wallis test were used. Vector graphics were created with Biorender.com (https://biorender.com/).

## Results

### Prostate epithelial-cell–expressed genes define four tumor clusters

We identified 1629 genes expressed by epithelial cell populations vs. all other cell types from single-cell and bulk RNA-seq data of human prostate-tissue samples (Supplementary Table S1, courtesy of Dr. Douglas Strand at UT Southwestern Medical Center) [14]. Initial consensus self- organizing map clustering of the TCGA-PRAD dataset using these 1593 genes (24 removed due to gene ID/symbol mismatching) suggested an optimal number of clusters in the range 2–5, but the absence of a plateau in consensus cumulative distribution function (CDF) plots implied that the population could not be cleanly separated (Supplementary Fig. S1a, b). Alternatively, we filtered samples by DNA purity (ABSOLUTE, CLONET purity values >0.5) and RNA purity (ISOpure, DeMix purity values >0.5), selecting 138 of 275 samples of purity data available (50.2%, Supplementary Table S2). Using this “pure” subset, we repeated consensus clustering and found four robust clusters (clusters A–D) with a minimal proportion of ambiguously clustered pairs (Supplementary Fig. S1c–f).

### Deconvolution analysis identify four transcriptomic subtypes of PCa

**Fig. 1:**
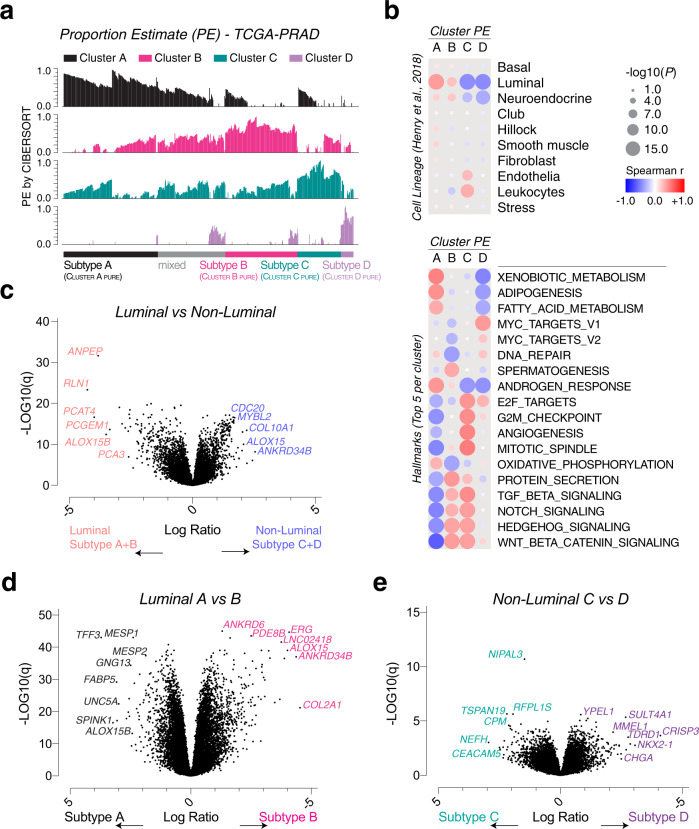
Transcriptomic characteristics of prostate cancer molecular subtypes. **a** Proportion estimate (PE) bar graphs of the four clusters and subtype assignment from the CIBERSORT deconvolution analysis of the TCGA-PRAD dataset. A sample was classified as “PURE” if PEmax >0.5 or as “mixed” if PEmax ≤0.5. **b** Correlation coefficient dot plot of ssGSEA scores of prostate cell lineage genesets, hallmark genesets and the cluster PE. Dot color = Spearman *r* value; Dot size = −log10(*P* value). Enrichment-volcano plot of gene expression comparisons between luminal clusters (A + B) and nonluminal clusters (C + D) (**c**), cluster A and B (**d**) or cluster C and D (**e**). Representative gene names are shown.

We generated gene signatures containing 1271 DEGs among the four clusters and calculated proportion estimates (PEs) of each cluster by running CIBERSORT deconvolution analysis (*P* < 0.05) (Fig. 1a, Supplementary Table S3). Single sample GSEA (ssGSEA) of the prostate cell lineage groups [14] and correlation analysis showed that cluster A and B were enriched by luminal cells, and cluster C by endothelia and immune cells (Fig. 1b, upper). Further analysis showed that cluster A enriched of adipogenesis and androgen response; cluster B enriched of spermatogenesis and protein secretion; cluster C enriched of G2M checkpoints, angiogenesis and mitotic spindle; cluster D enriched of Myc targets and DNA repair genesets (Fig. 1b, lower).

We assigned the samples to a subtype when the cluster PE was >0.5, and those with maximal PE ≤0.5 were designated “mixed”. By this definition, samples were classified as luminal subtypes A (*n* = 163, 30.0%), B (*n* = 141, 26.0%) and non-luminal subtypes C (*n* = 80, 14.7%), D (*n* = 23, 4.2%), or mixed (*n* = 136, 25.0%) (Fig. 1a). The luminal subtypes overexpressed *ANPEP* encoding *Alanine aminopeptidase* (*CD13*) and *RLN1* encoding *Relaxin*, both of which specifically expressed by mature luminal epithelial cells [14] (Fig. 1c). When comparing luminal subtypes A and B, luminal A overexpressed *SPINK1 and TFF3*, which are coexpressed in *ERG*-negative tumors [2, 19], whereas luminal B overexpressed *ERG* (Fig. 1d). When comparing non-luminal subtypes C and D, subtype C overexpressed conventional neuroendocrine PCa markers *CHGA* and *NKX2-1*, whereas subtype D overexpressed a recently discovered neuroendocrine PCa marker *CEACAM5* [20] (Supplementary Fig. S4f).

### Non-luminal subtypes are Aggressive Variant Prostate Cancers (AVPCs)

**Fig. 2:**
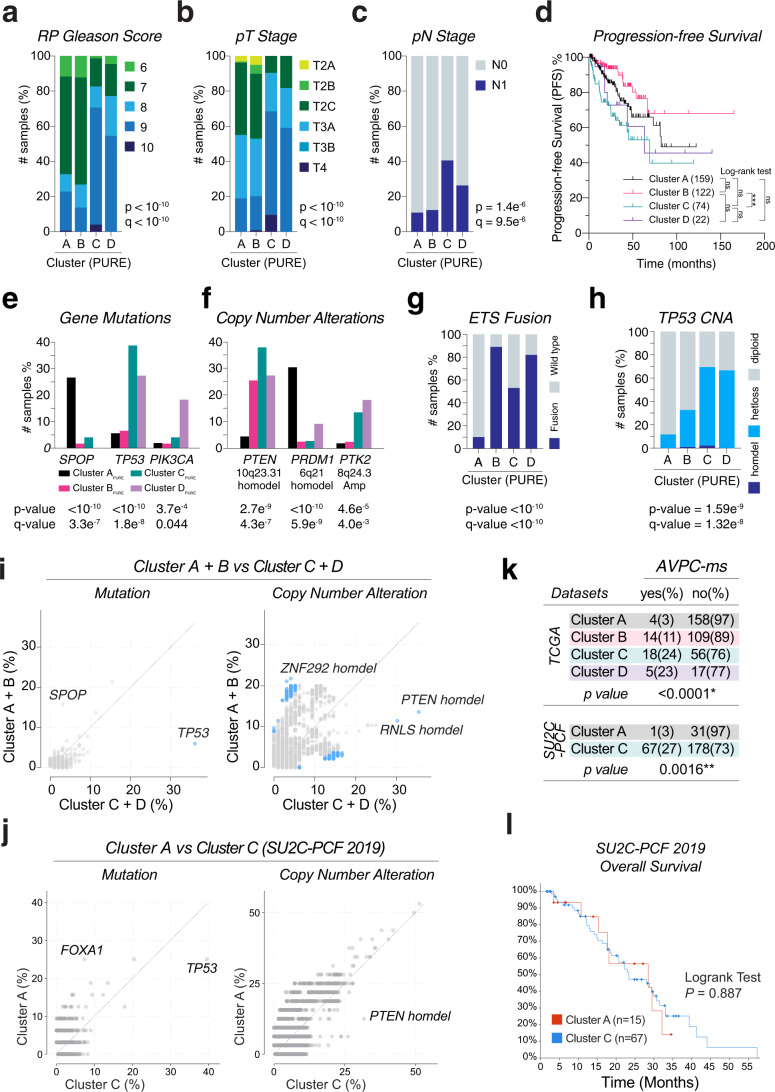
Pathologic and genomic characteristics of prostate cancer molecular subtypes. **a**–**c**. Cluster-wise distributions of radical prostatectomy (RP) Gleason score (**a**), pathologic T (pT) stage (**b**) and pN stage (**c**) from the TCGA-PRAD dataset. *P* values and *Q* values by chi-square test and Benjamini–Hochberg procedure. **d**. Kaplan–Meier Plot of Progression-free Survival. Survival data downloaded from cBio-Portal. P value by Log-rank test. **f**–**h**. Frequencies of SPOP, TP53, and PIK3CA mutations (**e**), copy-number alteration events (**f**), ETS-family fusions (ERG; ETV1, 4, 5, or 6) (**g**) and TP53 copy-number alteration (CNA) (**h**). *P* values and *Q* values by chi-square test and Benjamini–Hochberg procedure. **i**, **j**. Enrichment frequency scatter plot of genome-wide mutations and copy number alteration in between luminal subtype(s) vs. non-luminal subtype(s) of the TCGA-PRAD dataset (**i**) and the SU2C-PCF 2019 dataset (**j**). Dot color blue if *Q* value < 0.05 by chi-square test and Benjamini–Hochberg procedure. **k**. Frequency of AVPC molecular signature (AVPC-ms) in the clusters of TCGA dataset (upper) and SU2C-PCF dataset (lower). AVPC-ms = two or more of PTEN, TP53 or RB1 mutation/deletion. *P* values by chi-square test. **l**. Kaplan–Meier Plot of Progression-free Survival of the SU2C-PCF dataset. Survival data downloaded from cBio-Portal. *P* value by Log-rank test.

We compared the pathologic and genomic characteristics of the four subtypes using the cBioPortal’s group comparison function [21]. The non-luminal subtypes C and D were characterized by a higher Gleason score and more advanced T and N stages than subtypes A and B (Fig. 2a, b), reflected in relatively shorter progression-free survival (Fig. 2d). Subtype A was characterized by frequent *SPOP* mutation and Chr 6q21 homodeletion, and the absence of ETS family fusion. In contrast, subtypes B, C and D were characterized by ETS fusion and PTEN deletion. Further, Subtype C and D showed *TP53* mutation/loss of heterozygosity, *PIK3CA* mutation, and amplifications of Chr 8q24.3, which harbors *PTK2* or focal adhesion kinase (FAK) (Fig. 2e–h).

We applied the gene signature for deconvolution of three additional PCa datasets. The CPC- GENE 2017 dataset consists of localized non-indolent tumors (Gleason score 6–7, clinically organ-confined) [22]. The DKFZ 2018 dataset consists of tumors diagnosed in patients <55 years old [23], and the SU2C-PCF 2019 dataset consists of metastatic castration-resistant prostate cancer (mCRPC) [24]. Interestingly, the CPG-GENE localized tumors were consisted largely of subtype A (31%) and B (62%), while the DKFZ early-onset tumors were consisted mostly of subtype A (85%). The SU2C-PCF mCRPC tumors consisted of subtype A (12%) and C (88%). The distribution of Gleason score, tumor stage and genetic alteration events were similar to that of the TCGA dataset (Supplementary Fig. S2a–c). Subtype C of the SU2C-PCF mCRPC dataset showed a tendency of adrenal, hepatic and pulmonary metastases enriched (Supplementary Fig. S2c).

Because the non-luminal subtypes C and D are featured by advanced T/N stage, high Gleason score, *PTEN* and *TP53* alterations, they fit into the criteria of Aggressive Variant PCa (AVPC), which defined clinically by rapid progression after androgen deprivation, low PSA level relative to tumor burden, visceral metastasis, neuroendocrine markers or histology, and molecularly by two or more alterations of *PTEN*, *TP53* and *RB1* (AVPC molecular signature, AVPC-ms) [25]. Indeed, *TP53* mutation and *PTEN* deletion were the top significant genomic features of non-luminal subtypes (Fig. 2i, Supplementary Fig. S2d). Further analysis showed that 23–27% of non-luminal tumors had AVPC-ms, whereas only 3–11% of luminal tumors had AVPC-ms (Fig. 2j).

Interestingly, though, overall survival was not significantly different between subtype A and C in the SU2C-PCF dataset (Supplementary Fig. S2e).

### PCa subtypes intrinsic sensitivity to AR signaling inhibitors and docetaxel

To summary, both subtype A and B are enriched of luminal epithelial cell genes. Subtype A is enriched of adipogenesis and fatty acid metabolism genes, whereas subtype B is enriched of protein secretion and spermatogenesis. In this regards, we named the subtype A as **luminal A** (**A**dipogenic), and subtype B as **luminal S** (**S**ecretory). The luminal A subtype is further characterized by absence of ETS family fusion and high AR activity. The luminal S subtype has ETS fusion in similar ratio to the non-luminal subtypes C and D.

The most distinguishing features of subtype C and D from the luminal subtypes were the AVPC signatures – both molecularly (combined losses of *PTEN*, *TP53* or *RB1*), and pathologically (high Gleason scores and advanced T/N stages). Thereby, we considered them as **AVPCs**. Since subtype C is enriched of leukocyte genes and angiogenesis signature, we named it as **AVPC–I** (**I**mmune- infiltrative). Subtype D is instead characterized by Myc oncogene targets overexpression and chromosome 8q24 amplifications (where Myc is located), we named it as **AVPC-M** (**M**yc-active).

**Fig. 3:**
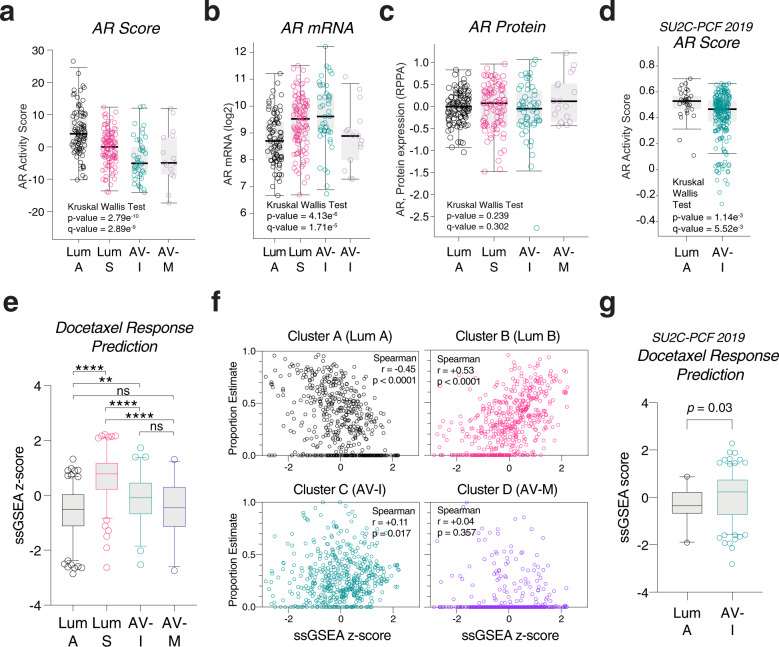
In silico drug sensitivity test of prostate cancer molecular subtypes. AR activity scores (**a**), mRNA expressions (**b**) and protein expressions (**c**) of the subtypes from the TCGA-PRAD dataset. One-way ANOVA and Kruskal–Wallis test. **d** AR activity scores of luminal A and AVPC-I subtypes from the SU2C-PCF dataset. **e** Pair-wise comparison of docetaxel response score among the subtypes. Dunn’s multiple comparisons test. ns not significant. *p < 0.05; **p < 0.01; ***p < 0.001; ****p < 0.0001. **f** Scatter plots of the four cluster PEs (Y-axis) and docetaxel response score of each sample. Spearman correlation coefficient and *p* values are shown in the box. **g** Comparison of docetaxel response score between luminal A and AVPC-I subtypes from the SU2C-PCF dataset. Mann–Whitney test.

Reduced AR transcriptional activity of tumor is a feature of AVPC, often reflecting by relative low PSA and can predict AR signaling inhibitor (ARI) resistance [25, 26]. To gauge the subtypes sensitivity to ARI, we used pre-calculated AR activity scores [3, 24]. AR score was highest in luminal A, followed by luminal B, and lowest in the two AVPC subtypes as expected (Fig. 3a). In contrast, *AR* mRNA levels were low in luminal A and AVPC-M (Fig. 3b), and AR protein levels were not different among the subtypes (Fig. 3c, d).

To differentiate PCa subtype-wise sensitivities to docetaxel, we used a modified version of an in silico drug-screening logic to predict sensitivity in cancer cell line samples based on their transcriptome data [27]. Briefly, gene sets were defined based on genes reported to positively or negatively correlate with tumor drug response. Drug response or sensitivity score in a sample is then computed by ssGSEA. Using this algorithm, we found that luminal S and AVPC-I are likely to be docetaxel responders, whereas luminal A and AVPC-M are likely to be docetaxel nonresponders (Fig. 3e, Supplementary Table S4). Correlation analysis was consistent with subtype-wise comparison, showing that cluster B (luminal S) and C (AVPC-I) proportion estimate (PE) positively correlate, and cluster A (luminal A) PE negatively correlates, with docetaxel responder score (Fig. 3f). The result of SU2C-PCF dataset was similar (Fig. 3g). The analysis also predicted that luminal A is likely to be resistant to paclitaxel (Supplementary Fig. S3a, b).

PCa docetaxel resistance has been associated with AR activation and loss of *KDM5D* (also known as *JARID1D*) expression, a histone demethylase enzyme gene [8, 28]. We found that *KDM5D* mRNA expression level was lowest in luminal A subtype (Supplementary Fig. 3c). For the AVPC-M subtype, the *in silico* drug screening suggested DNA damage-inducing purine analogues as potential drug of choice (Supplementary Fig. 3d and Supplementary Table S4).

### Serum PSA/PAP ratio predicts progression-free survival after docetaxel in mCRPC

**Fig. 4:**
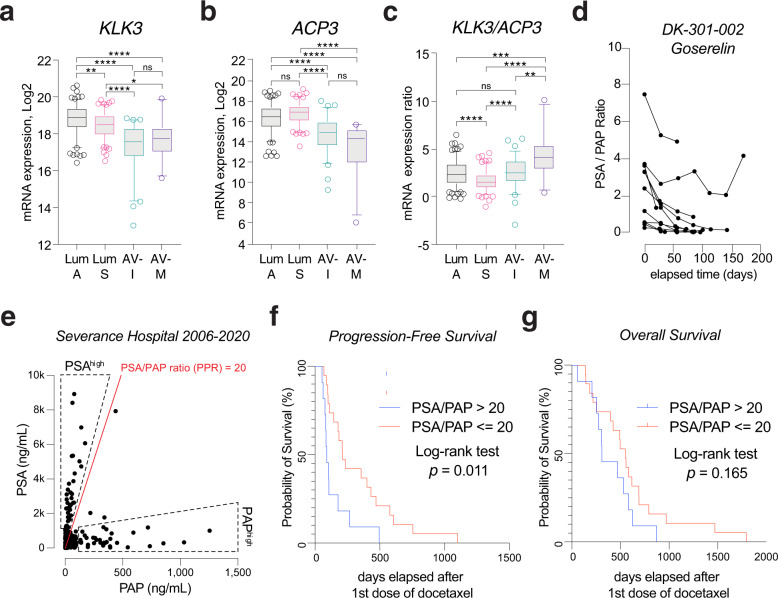
Serum PSA/PAP ratio predicts docetaxel response in mCRPC. KLK3 (**a**) and ACP3 (**b**) mRNA expression (log2 RSEM) levels among the subtypes from the TCGA-PRAD dataset. Dunn’s multiple comparisons test. ns not significant. *p < 0.05; **p < 0.01; ***p < 0.001; ****p < 0.0001. **c** KLK3/ACP3 mRNA expression ratio, calculated by subtracting ACP3 from KLK3 log2-transformed RSEM values. Dunn’s multiple comparisons test. ns not significant. *p < 0.05; **p < 0.01; ***p < 0.001; ****p < 0.0001. **d** Serum PSA/PAP ratio changes before and during goserelin injection in treatment-naïve PCa patients of DK-301-002 trial. **e** Scatter plot of serum PSA and PAP levels measured simultaneously in samples of prostate cancer patients of Severance Hospital from Jan 2006 to July 2020. Dotted area, upper: PSAhigh samples; dotted area, lower: PAPhigh samples. Red line = PSA/PAP ratio = 20. Kaplan–Meier plots of radiographic progression-free survival (**f**) and overall survival (**g**) of mCRPC patients following docetaxel-prednisone chemotherapy. Samples were divided by pretreatment serum PSA/PAP ratio >20 or ≤20. Log-rank test. Radiographic response by RESIST 1.1 criteria.

*KLK3* gene encoding prostate specific antigen (PSA) is an AR target gene incorporated in the AR activity score [3]. ETS fusion-negative and *SPOP*-mutated tumors are characterized by higher PSA than ETS fusion-positive tumors [29, 30]. As expected, *KLK3* mRNA level was highest in luminal A, followed by luminal S, and lowest in the two AVPC subtypes (Fig. 4a). However, preoperative serum PSA levels were not significantly different among the subtypes, either by average level or by distribution (Supplementary Fig. S4a). This is likely due to the variance of tumor burden across samples. When stratified by pT stage, pT2C luminal A tumors showed significantly higher PSA levels than pT2C luminal B (Supplementary Fig. S4b). In the mCRPC dataset, luminal A tumors serum PSA and *KLK3* mRNA levels were significantly higher than AVPC-I tumors (*p* < 0.01, Supplementary Fig. S4c, d). *KLK3* mRNA level was significantly higher in mCRPC enzalutamide responder group than nonresponder group, too (Supplementary Fig. S4e).

*ACP3* gene encodes prostatic acid phosphatase (PAP), an early generation PCa serum marker. The *ACP3* mRNA level was higher in luminal subtypes than the AVPC subtypes in the TCGA dataset (Fig. 4b). However, in the mCRPC datasets, the *ACP3* mRNA level was not significantly different between luminal A and AVPC-I, or enzalutamide responders and nonresponders (Supplementary Fig. S4d, e). The difference between TCGA dataset and the mCRPC datasets are the presence/absence of ongoing androgen deprivation therapy (ADT). Indeed, a clinical trial (DK-301) from our institution evaluating goserelin acetate 10.8-mg depot as first-line ADT in advanced PCa patients (*n* = 12) showed a gradual decline of PSA/PAP ratio following first-line ADT (Fig. 4d).

Based on the in silico drug sensitivity test and serologic characteristics, we hypothesized that the two PCa specific serum markers PSA and PAP combination can predict molecular subtypes and drug responses of mCRPC patients: the *KLK3*/ACP3 mRNA expression ratio is lowest in AVPC-M and lumina A subtypes that are predicted to be docetaxel-resistant (Fig. 4c). We reviewed 1052 serum PSA and PAP paired samples from 651 patients of our institution from 2006 to 2020 (Supplementary Fig. S4f, further details described in methods section), which revealed two subpopulations of PSA^high^ and PAP^high^ (Fig. 4e). We further examined 30 mCRPC cases who received docetaxel-predisone chemotherapy and eligible for radiographic progression and survival analysis. Their serum PSA/PAP ratio did not show a downward trend during docetaxel treatment (Supplementary Fig. 4g). From the earlier analysis, we estimated that 25–35% of the mCRPC patients would be classified as AVPC-M or luminal A subtypes, and divided the groups in to pretreatment PSA/PAP ratio >20 (*n* = 11, 36.7%) and ratio ≤20 (*n* = 19, 63.3%). Of note, the cut-off value (PSA/PAP ratio = 20) separated the PSA^high^ population from the rest in the scatter plot (Fig. 4e). Intriguingly, we found that the PSA/PAP ratio >20 group showed significant shorter progression-free survival than the PSA/PAP ratio ≤20 group (median survival: 91 days vs. 210 days, Log-rank test, *p* = 0.011). Hazard ratio (Mantel–Haenszel) was 3.4 (95% CI = 1.3–8.8). The overall survival did not show significant differences (median survival: 309 days vs. 551 days, Log-rank test, *p* = 0.165) (Individual sample data available in Supplementary Table S5).

## Discussion

In case of transcriptome-based molecular subtyping of breast cancer [31, 32], expression of a set of 494 breast tumor-cell–intrinsic genes was defined to overcome heterogeneity arising from the stroma [31]. Following this strategy, we used previously deposited prostate-tissue epithelial cell- lineage–specific gene expression profiles assessed by bulk and single-cell RNA sequencing [14, 15]. We discovered four transcriptomic subtypes of primary prostate adenocarcinoma – luminal A, luminal S, AVPC-I and AVPC-M (Table 1 summarized their characteristics). Our classification partly overlapped with earlier findings from multi-omics-based or marker-based clustering approaches [1–3, 19]. However, subtype definitions were not absolute, resulting in classification of ~25% of tumors as mixed. Strikingly, *KLK3* and *ACP3* mRNA expression levels, encoding PSA and PAP, respectively, showed potential to identify subtypes; this was further supported by serum PSA and PAP levels measured before prostatectomy or docetaxel chemotherapy.

**Table 1:** Characteristics of the prostate cancer molecular subtypes.

Molecular subtype	Luminal A	Luminal S	AVPC-I	AVPC-M
*Consensus cluster (Prevalence*^a^*)*	A (30.0%)	B (26.0%)	C (14.7%)	D (4.2%)
*Transcriptomic characteristics*	Luminal-like adipogenic	Luminal-like secretory	Immune, angiogenic cell cycle-active	Myc-active DNA-repairing
*Genomic characteristics*	*ETS* fusion (−) *SPOP*-mut (+) Chr6q del (+)	*ETS* fusion (+) *PTEN* del (+) *TP53* mut/del (−)	*ETS* fusion (+) *PTEN* del (+) *TP53* mut/del (+) *PIK3CA* mut (−)	*ETS* fusion (+) *PTEN* del (+) *TP53* mut/del (+) *PIK3CA* mut (+)
*AVPC-ms*^a^	3%	11%	24%	23%
*Serum PSA/PAP ratio (predicted)*	High	Low	Intermediate	High
*Docetaxel response*	Resistant	Sensitive	Sensitive	Resistant
*AR inhibitor response*	Sensitive	Sensitive	Resistant	Resistant
*Potential therapy*	AR inhibitors	AR inhibitors +Taxanes	Taxanes +Immunotherapy	DNA-damaging agents (Platinum, Purine analogues)

^a^Pure samples only, measured in TCGA-PRAD dataset. AVPC-ms = two or more alterations of *TP53*, *PTEN* or *RB1*.

Identification of cancer molecular subtypes has deepened our understanding of cancer biology and clinical implications, including therapeutic target identification. In breast cancer docetaxel adjuvant chemotherapy was not beneficial in the luminal A population or in patients with ER- positive and HER2-negative cancers [33–35]. We claim that such findings can be translated to PCa. Our analysis suggests that luminal A subtype, with the strongest AR activity, should undergo treatment with the new AR target agents and avoid taxanes if diagnosed in advanced stage [36].

Encouragingly, data from recent molecular profiling of mCSPCs support that the *SPOP*-mutated tumors are less likely to become castration-resistant [37]. In contrast, *TP53* inactivation, a distinguishing feature of AVPC subtypes from the luminal tumors, was predictive of abiraterone and enzalutamide outcomes in mCRPCs [38]. For the AVPC-M subtype which predicted to be resistant to both docetaxel and AR signaling inhibitors, DNA damage-inducing agents (purine analogues) might be tried. Indeed, clinical trials showed that AVPC-ms (+) tumors can benefit from DNA-damaging platinum-based chemotherapies in addition to cabazitaxel [39].

The established PCa serum biomarker combination of PSA and PAP may be useful to predict the transcriptomic subtype and docetaxel sensitivity at an advanced stage. Reports shows that the 5- year survival rate was significantly lower in metastatic cancer patients with a low PSA/PAP ratio than with a high ratio (24% vs. 48%, *P* = 0.002) [40]. In localized tumors, elevated PAP before treatment has been regularly identified as a significant prognostic factor following definitive therapies [41–44]. Although the benefit of docetaxel is repeatedly seen in mCRPC, adjunct docetaxel therapy is not superior to androgen deprivation therapy alone in high-risk cancer with rising PSA only [45]. We postulate that patients with rising PSA and PAP together (low PSA/PAP ratio) may be the suitable candidates to test the benefit of early docetaxel treatment. In contrast, patients with rising PSA but not PAP (high PSA/PAP ratio) are most likely of luminal A or AVPC-M subtypes, which are predicted to be insensitive to docetaxel. The PAP protein is also a target antigen of therapeutic vaccine Sipleucel-T [46]. We found a substantial variability of PAP expression per tumor cell, which suggest that the benefit of immunotherapy may be subtype-specific, like as ARI and docetaxel. Indeed, a post-hoc analysis found that lower baseline PSA is associated with a greater overall survival benefit [47]. Together, we propose to measure serum PAP in combination with PSA and consider their ratio as tumor cell subtype marker in settings of systemic therapy. While the individual markers expression may correlate with the progression during therapy, the PSA/PAP ratio maybe a specific marker of tumor cell subtype, since the variation of tumor burden is subtracted out in the calculation. It is possible that free PSA and PAP combination maybe a better markers, though, considering the biological half-life of PSA (2-3 days), free PSA and PAP (1.1–2.6 h) [48].

**Fig. 5:**
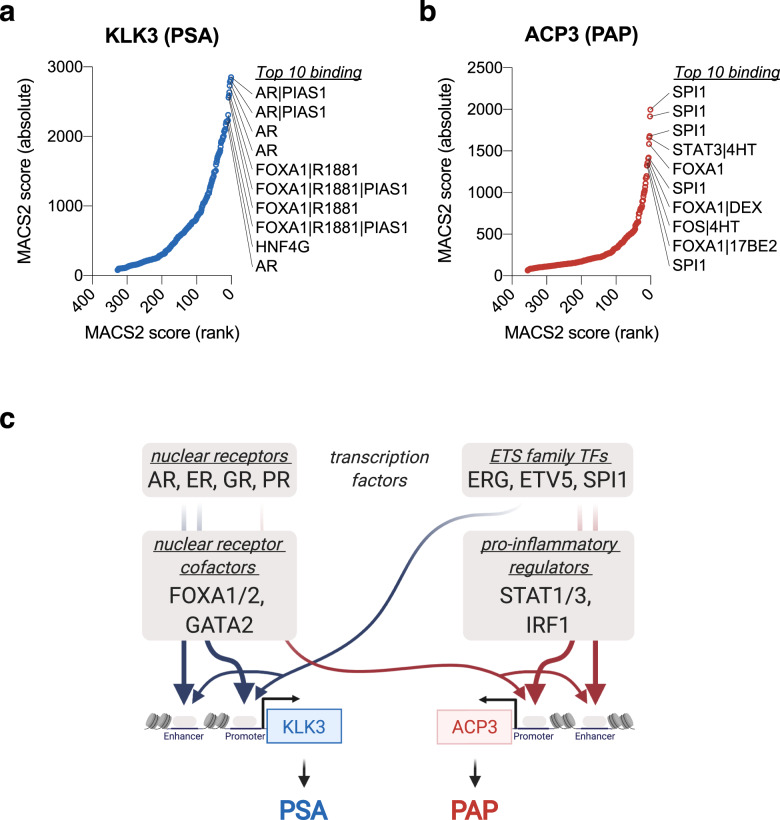
Upstream regulators of KLK3 and ACP3 genes expression. KLK3 (**a**) and ACP3 (**b**) genes promoters and enhancers public ChIP-seq data integrative analysis (original data available from the Signaling Pathways. Each transcriptional factor binding site was identified by the Model-based Analysis of ChIP-seq (MACS2) algorithm and ranked by the MACS2 score. Top 10 TFs are listed. **c** a schematic diagram of transcriptional regulators of KLK3 and ACP3 genes. Note that while both genes are regulated by AR and AR cofactors, ACP3 is (in addition) regulated by pro-inflammatory factors such as STAT1/3 and IRF1.

Both KLK3 (coding PSA) and ACP3 (coding PAP) genes are regulated by AR. An integrative analysis on their gene promoters and enhancers public ChIP-seq data (available from the Signaling Pathways Project http://signalingpathways.org) indicate that ACP3 gene is regulated also by ETS family transcription factors (SPI1, ERG) and inflammation-related factors (STAT1, STAT3) (Fig. 5a, b, Supplementary Table S6). This might be the underlying mechanism that PSA/PAP ratio in general decreases following androgen deprivation therapy (Fig. 4d). In other words, PSA/PAP ratio may reflect the activity of alternative signaling pathways (ETS family, STATs) that leads to early onset castration-resistance (Fig. 5c).

In addition to the fact that SPOP-driven prostate tumors (Luminal A) and ERG-driven tumors (Luminal S and AVPCs) exist in mutually exclusive manner, our analysis support that the AVPCs mostly arise in ERG-driven tumors by losses of PTEN and p53. ERG activation coordinate with PTEN loss in prostate cancer progression, and it is likely that loss of p53 on top of ERG/PTEN loss promote androgen-independent tumor growth and metastasis. These are shared characteristic of AVPC-I and AVPC-M, and imply that the AVPCs harbor significant chromosomal instability that potentially be associated with microtubule stabilizer’s anti-tumor mechanism. We further speculate that compared to AVPC-I, AVPC-M have less vascularization, slow in cell cycle, and frequent genetic mutations of PI3K-Akt-mTOR pathway that promotes resistance to taxanes.

Our finding does not contradict earlier reports that *ERG* induces taxane resistance in CRPC [49]. Rather, it underscores the importance of radiographic and clinical responses over PSA response in mCRPC cases, where increasing numbers of PSA-low neuroendocrine–like cancers are seen. We argue that ETS-fusion tumors can be subdivided - luminal S, AVPC-I and AVPC-M, and the taxane-resistance and castration-resistance might be dependent on molecular contexts such as combined losses of *PTEN*, *TP53* or *PIK3CA* associated with ETS fusion [5–7].

Interpretation of our data is limited due to the study’s retrospective design and unplanned subset analysis. For instance, serum PAP data was not available for the TCGA or the SU2C-PCF dataset. For docetaxel response analysis in our cohort, we used pre-chemotherapy serum PSA and PAP levels measured after long-term androgen deprivation. Analysis of the magnitude of the decrease in those markers suggest that the PSA/PAP ratio may change during androgen deprivation. A further prospective trial is warranted, particularly in metastatic castration-sensitive or non-metastatic castration-resistant settings, where the benefit of molecular subtyping and tailored therapies can be maximized. Molecular profiling of metastatic tumors is still a challenge because of difficulty in acquiring the representative tumor samples. Blood sampling of the established serum markers PSA, PAP, together with analysis of circulating tumor DNA may serve as a noninvasive marker combination sufficient to guide treatment choices.

Collectively, we present a novel PCa molecular classification system, connecting the previous efforts of classifying PCa to an unbiased, biology-oriented and clinically-relevant subtypes.

## Supplementary information

Supplementary Figure legends

Supplementary Figure S1

Supplementary Figure S2

Supplementary Figure S3

Supplementary Figure S4

Supplementary Table 1

Supplementary Table 2

Supplementary Table 3

Supplementary Table 4

Supplementary Table 5

Supplementary Table 6

## Data Availability

All data will be provided upon request.
