# Supplementary material for: Prostate epithelial genes define therapy-relevant prostate cancer molecular subtype
Source: Prostate Cancer Prostatic Dis. 2021 Apr 26;24(4):1080–92. doi: 10.1038/s41391-021-00364-x (PMC8616761; doi:10.1038/s41391-021-00364-x)
Supplement: Supplementary file 1 — Supplementary Figure legends [file 41391_2021_364_MOESM1_ESM.docx]

**SUPPLEMENTARY FIGURE legends**

**Supplementary Figure S1. Consensus Clustering of The Cancer Genome Atlas Prostate Adenocarcinoma**

**(TCGA-PRAD) RNA-Seq Data**

(a, b) Consensus Clustering of TCGA-PRAD RNA-Seq Data: (a) Consensus cumulative distribution function (CDF) plots of K=2 to K=15. (b) Delta area plot showing the proportion increase in area under the CDF curve comparing K and K-1. (c, d) Consensus Clustering of TCGA-PRAD RNA-Seq Data after filtering by RNA and DNA purity score: (c) Consensus CDF plots of K=2 to K=15. (d) Delta area plot showing the proportion increase in area under the CDF curve comparing K and K-1. (e) Proportion of ambiguously clustered pairs (PAC) from panel c, represented by ΔCDFK (CDFK[index value 0.9]-CDFK [index value 0.1]). Arrow indicates minimal PAC at K=4. (f) Heatmaps of clustered consensus matrices of K=2 to K=5.

**Supplementary Figure S2. Application of Classification in Additional Prostate Cancer Datasets**

(a-c) Radical prostatectomy (RP) Gleason score, pT stage distributions, and frequencies of ETS-family fusions and gene mutations among clusters identified in three additional prostate cancer datasets. (a) The CPC-GENE 2017 dataset. (b) The DKFZ 2018 dataset. (c) The SU2C-PCF 2019 dataset. For SU2C-PCF dataset, samples of concordant deconvolution analysis results between RNA-Seq Poly-A and Capture were included. Tumors with neuroendocrine histologic features were excluded for analysis. Bar = Geometric mean with 95% CI. (d) Enrichment frequency scatter plot of genome-wide mutations and copy number alteration in between luminal subtypes vs. non-luminal subtypes. (e) Kaplan-Meier Plot of Overall survival of the SU2C-PCF dataset. Survival data downloaded from cBioPortal. P value by Log-rank test.

**Supplementary Figure S3. In silico Drug Sensitivity Test comparing the subtypes**

(a) Pair-wise comparison of paclitaxel sensitivity score among the four subtypes. Dunn’s multiple comparisons test. ns=not significant. *p<0.05; **p<0.01; ***p<0.001; ****p<0.0001. (b) Scatter plots of the four cluster PEs (Y-axis) and paclitaxel sensitivity score of each sample. Spearman correlation coefficient and p-values are shown in the box. (c) KDM5D mRNA expressions of the four subtypes from the TCGA-PRAD dataset. Dunn’s multiple comparisons test. ns=not significant. *p<0.05; **p<0.01; ***p<0.001; ****p<0.0001. (d) Volcano plot of enrichment score from in silico drug sensitivity screening against AVPC-M subtype. DNA damaging purine analogues among the top ranks are shown.

P value by Spearman correlation test.

**Supplementary Figure S4. In silico Paclitaxel Drug Sensitivity Test comparing the subtypes**

(a) Preoperative (radical prostatectomy, RP) serum PSA levels presented in box plot (5%–95%, upper) and stacked bar chart (class interval: 10 ng/mL, 20 ng/mL, lower). Multiple comparison assessed by Kruskal-Wallis test. (b) Luminal A and luminal S subtypes preoperative serum PSA levels, stratified by pT stage. Multiple t-test, Holm-Sidak method, without assuming a consistent standard deviation. (c) Serum PSA levels of luminal A and AVPC-I subtypes from the SU2C-PCF 2019 dataset. P value by Mann-Whitney test. (d) KLK3 and ACP3 mRNA expression levels of luminal A and AVPC-M subtypes from the SU2C-PCF mCRPC dataset. (e) KLK3 and ACP3 mRNA expression levels of metastatic tissues from mCRPC patients receiving enzalutamide. Samples divided into responders and nonresponders by PSA50 response (PSA decline of ≥50% at 12 wk compared with baseline) RNA-Seq data from Alumkal et al. (Alumkal et al., PNAS, 2020). (f) Scatter plot of two prostatic acid phosphatase (A.P.) test result values measured simultaneously in samples of prostate cancer patients of Severance Hospital from Jan 2006 to July 2020. Line = Linear Regression Analysis. (g) Serum PSA/PAP ratio changes before and during goserelin injection in docetaxel-prednisone chemotherapy in mCRPC patients.
