# Supplementary material for: Prostate epithelial genes define therapy-relevant prostate cancer molecular subtype
Source: Prostate Cancer Prostatic Dis. 2021 Apr 26;24(4):1080–92. doi: 10.1038/s41391-021-00364-x (PMC8616761; doi:10.1038/s41391-021-00364-x)
Supplement: Supplementary file 2 — Supplementary Figure S1 [file 41391_2021_364_MOESM2_ESM.pdf]

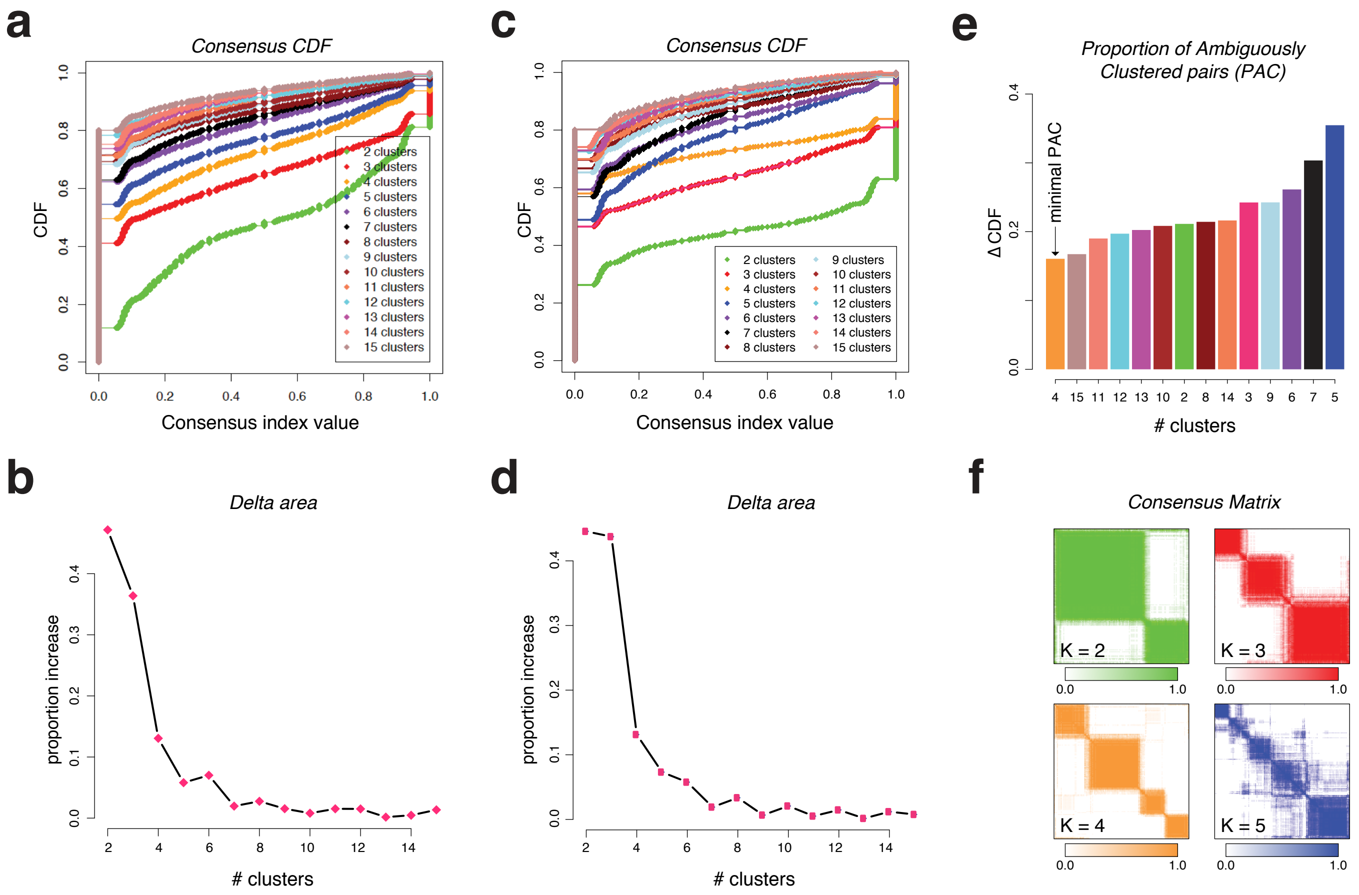

Supplementary Figure S1. Consensus Clustering of The Cancer Genome Atlas Prostate Adenocarcinoma (TCGA-PRAD) RNA-Seq Data

(a, b) Consensus Clustering of TCGA-PRAD RNA-Seq Data: **(a)** Consensus cumulative distribution function (CDF) plots of K=2 to K=15. **(b)** Delta area plot showing the proportion increase in area under the CDF curve comparing K and K-1. **(c, d)** Consensus Clustering of TCGA-PRAD RNA-Seq Data after filtering by RNA and DNA purity score: **(c)** Consensus CDF plots of K=2 to K=15. **(d)** Delta area plot showing the proportion increase in area under the CDF curve comparing K and K-1. **(e)** Proportion of ambiguously clustered pairs (PAC) from panel c, represented by ΔCDF_K (CDF_K[index value 0.9]-CDF_K [index value 0.1]). Arrow indicates minimal PAC at K=4. **(f)** Heatmaps of clustered consensus matrices of K=2 to K=5.
