# Supplementary material for: Prostate epithelial genes define therapy-relevant prostate cancer molecular subtype
Source: Prostate Cancer Prostatic Dis. 2021 Apr 26;24(4):1080–92. doi: 10.1038/s41391-021-00364-x (PMC8616761; doi:10.1038/s41391-021-00364-x)
Supplement: Supplementary file 3 — Supplementary Figure S2 [file 41391_2021_364_MOESM3_ESM.pdf]

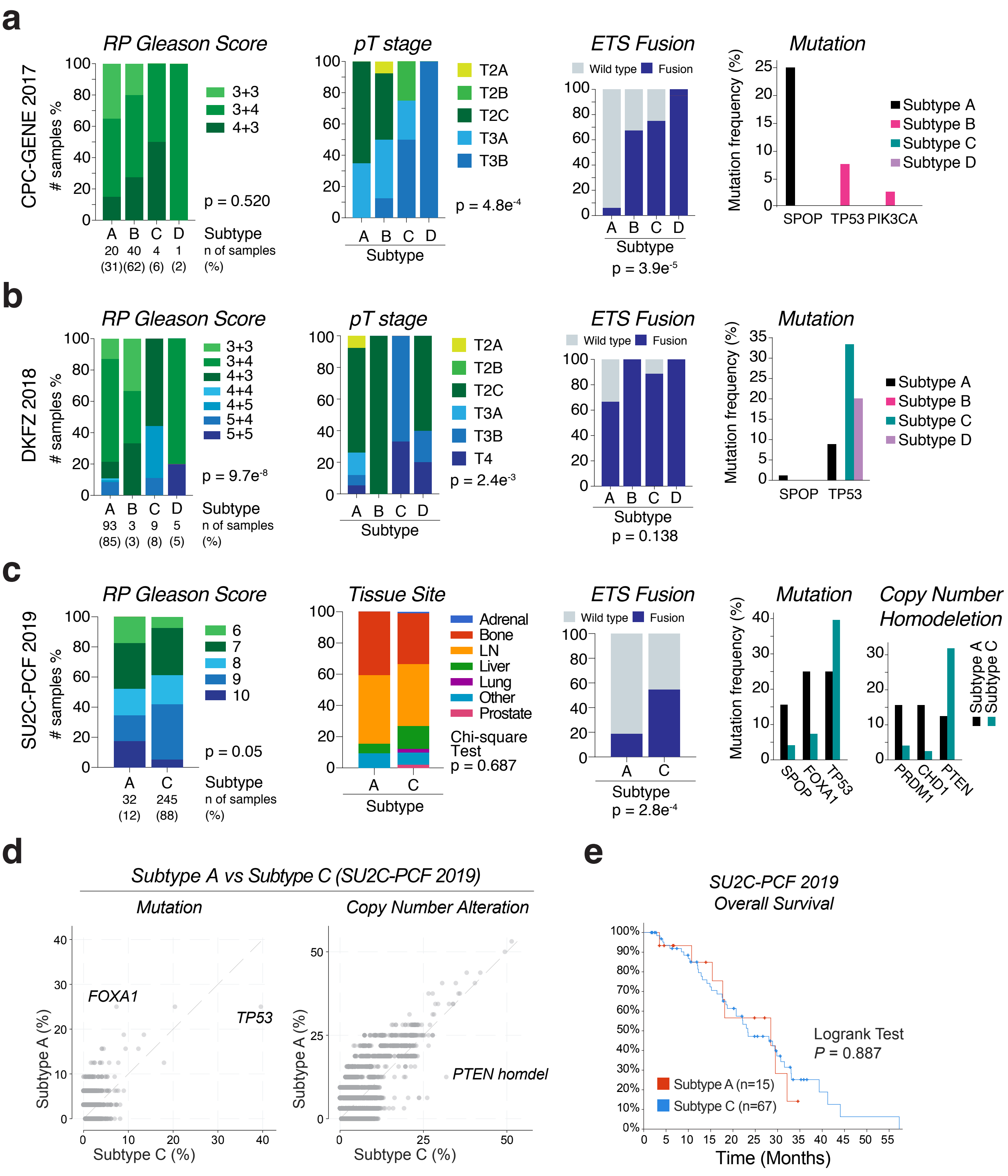

Supplementary Figure S2. Application of Classification in Additional Prostate Cancer Datasets
(a-c) Radical prostatectomy (RP) Gleason score, pT stage distributions, and frequencies of ETS-family fusions and gene mutations among clusters identified in three additional prostate cancer datasets. **(a)** The CPC-GENE 2017 dataset. **(b)** The DKFZ 2018 dataset. **(c)** The SU2C-PCF 2019 dataset. For SU2C-PCF dataset, samples of concordant deconvolution analysis results between RNA-Seq Poly-A and Capture were included. Tumors with neuroendocrine histologic features were excluded for analysis. Bar = Geometric mean with 95% CI. **(d)** Enrichment frequency scatter plot of genome-wide mutations and copy number alteration in between luminal subtypes vs. non-luminal subtypes. **(e)** Kaplan-Meier Plot of Overall survival of the SU2C-PCF dataset. Survival data downloaded from cBioPortal. P value by Log-rank test.
