# Supplementary material for: Prostate epithelial genes define therapy-relevant prostate cancer molecular subtype
Source: Prostate Cancer Prostatic Dis. 2021 Apr 26;24(4):1080–92. doi: 10.1038/s41391-021-00364-x (PMC8616761; doi:10.1038/s41391-021-00364-x)
Supplement: Supplementary file 4 — Supplementary Figure S3 [file 41391_2021_364_MOESM4_ESM.pdf]

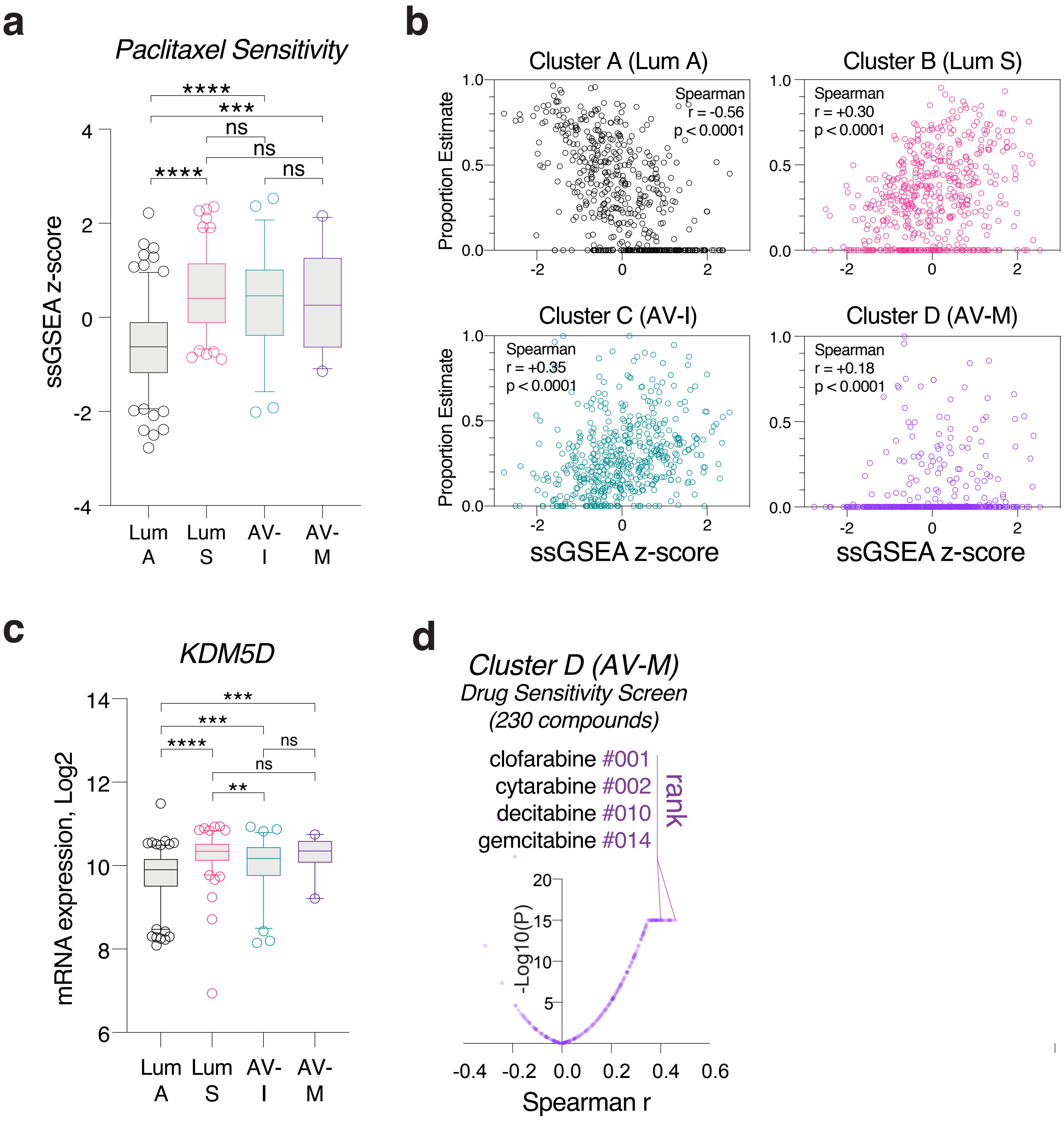

Supplementary Figure S3. In silico Drug Sensitivity Test comparing the subtypes

(a) Pair-wise comparison of paclitaxel sensitivity score among the four subtypes. Dunn's multiple comparisons test. ns=not significant. *p<0.05; **p<0.01; ***p<0.001; ****p<0.0001. **(b)** Scatter plots of the four cluster PEs (Y-axis) and paclitaxel sensitivity score of each sample. Spearman correlation coefficient and p-values are shown in the box. **(c)** KDM5D mRNA expressions of the four subtypes from the TCGA-PRAD dataset. Dunn's multiple comparisons test. ns=not significant. *p<0.05; **p<0.01; ***p<0.001; ****p<0.0001. **(d)** Volcano plot of enrichment score from in silico drug sensitivity screening against AVPC-M subtype. DNA damaging purine analogues among the top ranks are shown. P value by Spearman correlation test.
