# Supplementary material for: Prostate epithelial genes define therapy-relevant prostate cancer molecular subtype
Source: Prostate Cancer Prostatic Dis. 2021 Apr 26;24(4):1080–92. doi: 10.1038/s41391-021-00364-x (PMC8616761; doi:10.1038/s41391-021-00364-x)
Supplement: Supplementary file 5 — Supplementary Figure S4 [file 41391_2021_364_MOESM5_ESM.pdf]

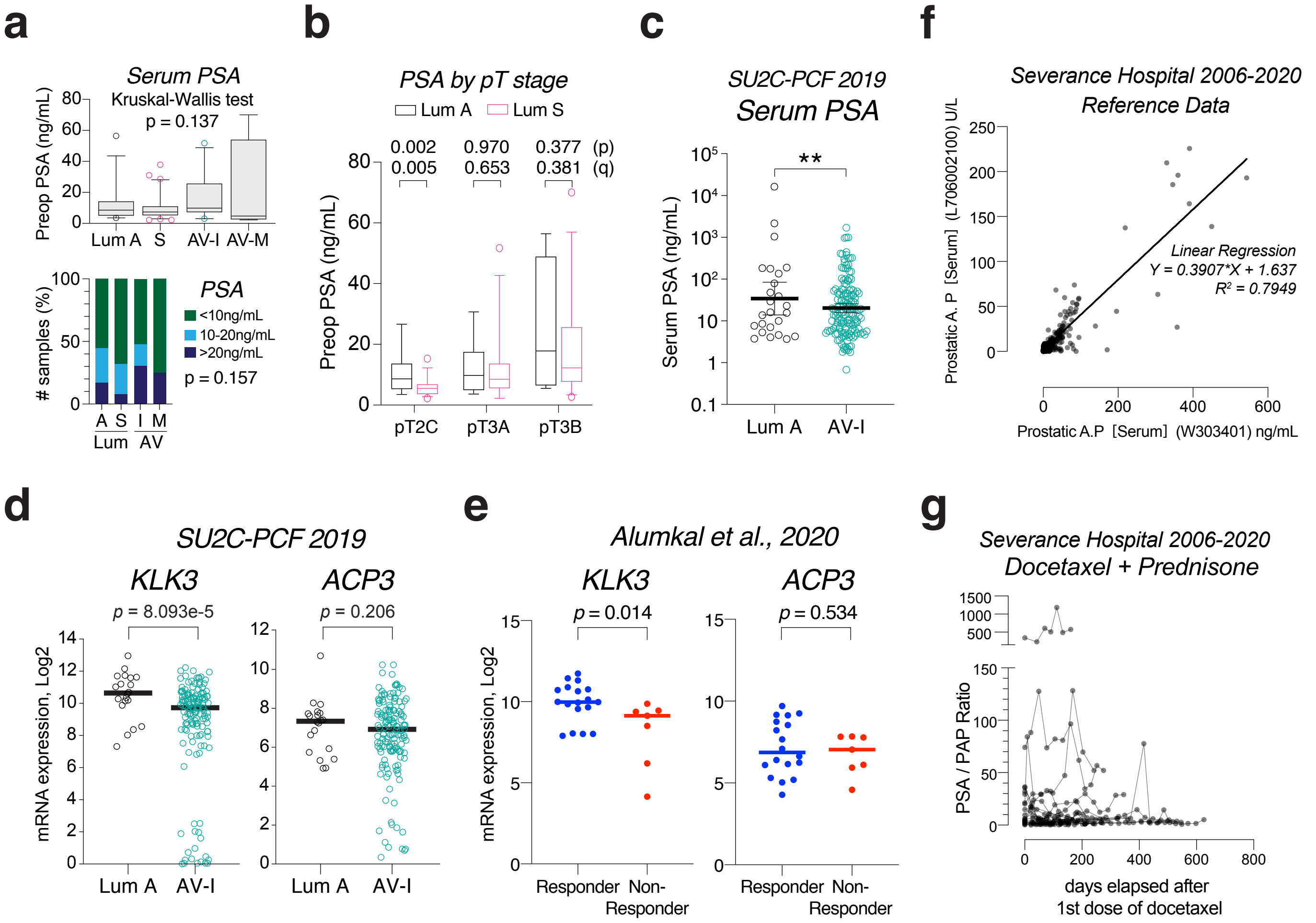

Supplementary Figure S4. In silico Paclitaxel Drug Sensitivity Test comparing the subtypes
(a) Preoperative (radical prostatectomy, RP) serum PSA levels presented in box plot (5%–95%, upper) and stacked bar chart (class interval: 10 ng/mL, 20 ng/mL, lower). Multiple comparison assessed by Kruskal-Wallis test. **(b)** Luminal A and luminal S subtypes preoperative serum PSA levels, stratified by pT stage. Multiple t-test, Holm-Sidak method, without assuming a consistent standard deviation. **(c)** Serum PSA levels of luminal A and AVPC-I subtypes from the SU2C-PCF 2019 dataset. P value by Mann-Whitney test. **(d)** KLK3 and ACP3 mRNA expression levels of luminal A and AVPC-M subtypes from the SU2C-PCF mCRPC dataset. **(e)** KLK3 and ACP3 mRNA expression levels of metastatic tissues from mCRPC patients receiving enzalutamide. Samples divided into responders and nonresponders by PSA50 response (PSA decline of ≥50% at 12 wk compared with baseline) RNA-Seq data from Alumkal et al. (Alumkal et al., PNAS, 2020). **(f)** Scatter plot of two prostatic acid phosphatase (A.P.) test result values measured simultaneously in samples of prostate cancer patients of Severance Hospital from Jan 2006 to July 2020. Line = Linear Regression Analysis. **(g)** Serum PSA/PAP ratio changes before and during goserelin injection in docetaxel-prednisone chemotherapy in mCRPC patients.
